# Influenza C Virus Infection in Children, Spain

**DOI:** 10.3201/eid1210.051170

**Published:** 2006-10

**Authors:** Cristina Calvo, Maria Luz García-García, Miriam Centeno, Pilar Pérez-Breña, Inmaculada Casas

**Affiliations:** * Severo Ochoa Hospital, Madrid, Spain;; † Instituto de Salud Carlos III, Madrid, Spain

**Keywords:** Influenza C virus, infants, respiratory infections, bronchiolitis, wheezing

**To the Editor:** Influenza viruses cause serious respiratory illness, particularly in infants <24 months of age (*1*). Despite serologic studies of French adults that showed an influenza virus seroprevalence of 60%–70%, influenza C infections have rarely been described (*2*). Given the technical difficulties involved in isolating influenza C virus in cell cultures, diagnosis is made only in certain laboratories. Detection of viral genome by reverse transcription (RT)–PCR in nasopharyngeal aspirates allows etiologic diagnosis of these infections (*3*). Mild upper respiratory infections in adults and adolescents are attributed to this virus (*4**,**5*). Some cases of lower respiratory infections have also been described in children (*6*).

A prospective study was conducted from September 1999 through July 2003. We determined the incidence and clinical manifestations associated with influenza C infection in all children <24 months of age admitted to Severo Ochoa Hospital in Madrid, Spain, with respiratory tract infections both with and without fever. All patients were evaluated by an attending physician. The study was approved by the Fondo de Investigaciones Sanitarias Committee of Spain.

Specimens of nasopharyngeal aspirates were obtained from each patient on admission (Monday to Friday) and sent to the Respiratory Virus Laboratory at the National Microbiology Center in Madrid for virologic studies. Specimens were processed within 24 hours of collection.

A multiplex RT-PCR was used for direct detection of respiratory syncytial virus A (RSV-A), RSV-B, adenoviruses, and influenza A, B, and C viruses in all nasopharyngeal samples, as previously described (*7*). Primers were specific for the nucleoprotein gene segment of influenza virus, the fusion gene of RSV, and the hexon gene of adenoviruses.

An internal amplification control was included in the reaction mixture to exclude false-negative results caused by specimen inhibitors or extraction failure. Given the high sensitivity of nested PCR, precautions were taken to prevent reactions from being contaminated with previously amplified product, as well as to protect target RNA or DNA from other specimens and controls. All procedures were performed in laboratory safety cabinets at locations different from those where amplified products were analyzed. Detection levels of 0.1 and 0.01 50% tissue culture infectious doses of influenza A and B viruses and 1–10 molecules of cloned amplified products of influenza C virus, RSV-A, RSV-B, and adenovirus serotype 1 were achieved.

A total of 706 hospitalized infants were enrolled in the study; 496 specimens were positive for virus (76.1% were RSV). Thirty children were infected with influenza virus (4.3% of all respiratory infections and 6% of all confirmed viral infections). Six patients had confirmed influenza C virus infections. Three of them had co-infections, 2 with RSV and 1 with adenovirus. Clinical characteristics of these 6 patients are shown in the Table. Although clinical characteristics for 24 influenza A virus infections were similar to those for influenza C virus infections (no influenza B virus was identified), statistical analysis was not conducted because of small sample size.

**Table:** Characteristics of 6 children with influenza C virus infections, Spain 1999–2003*

	Patient					
Characteristic	1	2	3	4	5	6
Age, mo.	12	15	24	3.5	11	7
Admission date	Jan 2000	Feb 2000	Mar 2000	Jan 2002	Nov 2002	Feb 2003
Sex	F	M	M	F	M	F
Temperature >38.5°C	Yes	Yes	Yes	Yes	Yes	Yes
Fever duration, d	15	5	3	10	2	2
Chest radiograph	Infiltrate	Normal	Infiltrate	Normal	Normal	Infiltrate
Diagnosis	Wheeze	Wheeze	Pneumonia	Bronchiolitis	Wheeze	Wheeze
O_2_ saturation <95%	No	Yes	No	No	Yes	Yes
Days in hospital	9	8	2	2	2	13
Coinfection	No	No	Adenovirus	RSV-B	RSV-B	No
Diarrhea	Yes†	No	Yes‡	No	No	Yes†

*RSV-B, respiratory syncytial virus B. †Rotavirus in feces. ‡Adenovirus in feces.

Influenza virus infections are a major cause of hospitalization and illness in young children, particularly those <2 years of age (*1*). Influenza A virus infections are more common than influenza B virus infections (75% vs 25%) (*8*). Our results indicate that influenza C virus is present in infants hospitalized with respiratory infections.

In contrast to data for adults, in whom influenza C virus infection is associated with mild upper respiratory infections (*5*), our study showed that this infection in infants may be associated with illness severe enough to require hospitalization. Clinical symptoms of influenza C virus infection in our patients, such as high fever and respiratory symptoms, were similar to those described for infections with influenza A and B viruses (*1**,**8**,**9*). Nonrespiratory symptoms such as fever or diarrhea have often been associated with influenza A and B virus infections (*9*). Three patients with influenza C virus infections had diarrhea; 2 had rotavirus and adenovirus detected in feces, and 1 had rotavirus detected in feces. However, the high incidence of co-infections makes clarifying the role of influenza C virus as a causative agent in these conditions difficult.

Few studies have investigated influenza C virus infection in children. Katagiri et al. (*10*) described an outbreak characterized by fever and symptoms of upper respiratory infections in workers and children at a children's home. The largest pediatric study reported was that of Moriuchi et al. (*6*) of 20 cases of influenza C virus infection in children <15 years of age compiled in 2 years in a hospital setting among both inpatients and outpatients. They found clinical results similar to ours (lower respiratory infections). However, we could not make any conclusions about influenza C virus infections in nonhospitalized children.

Influenza C virus infection in hospitalized infants is responsible for a clinical condition with high fever and respiratory symptoms severe enough to require hospitalization. This virus should be studied in respiratory infections in hospitalized infants to further clarify its role.
